# Draft genome sequence of a multidrug-resistant *Mammaliicoccus sciuri* BAU-MFRK-Mam-2025 strain isolated from phuchka samples in Mymensingh, Bangladesh

**DOI:** 10.1128/mra.01210-25

**Published:** 2025-12-23

**Authors:** Prakash Chandra Shil, Md Abdullah Evna Hasan, Maysha Islam, Layla Yasmin, Najmun Nahar Popy, Md. Bahanur Rahman, Mohammad Ferdousur Rahman Khan

**Affiliations:** 1 Department of Microbiology and Hygiene, Faculty of Veterinary Science, Bangladesh Agricultural University 54492, Mymensingh, Bangladesh; University of Manitoba, Winnipeg, Canada

**Keywords:** draft genome, *Mammaliicoccus sciuri*, phuchka, multidrug resistance, resistance gene

## Abstract

We report the draft genome sequence of *Mammaliicoccus sciuri* strain BAU-MFRK-Mam-2025 isolated from phuchka samples in Mymensingh, Bangladesh. The 2.78 Mb genome (GC 32.8%, genome coverage 50×) harbors multiple antibiotic resistance genes, underscoring its potential threat to food safety, antimicrobial resistance dissemination, and public health.

## ANNOUNCEMENT

*Mammaliicoccus sciuri* is a coagulase-negative, gram-positive, opportunistic zoonotic bacterium found in livestock, companion animals, and humans (1, 2). It has increasingly been reported in food environments and is associated with wound infections, urinary tract infections, and bacteremia (3, 4). Of particular concern is its multidrug resistance (MDR) to β-lactams, aminoglycosides, macrolides, and tetracyclines (5, 6), highlighting the need for robust genomic surveillance to mitigate public health risks. Here, we present the draft genome sequence of a multidrug-resistant *Mammaliicoccus sciuri* isolated from phuchka samples.

In January 2024, phuchka samples, a popular South Asian street food consisting of a hollow fried shell filled with a spicy mixture and flavored water, were collected aseptically from vendors at Bangladesh Agricultural University campus, Mymensingh (24.7170°N, 90.4266°E), Bangladesh following previously described approaches (7). Approximately 10 g of phuchka sample was collected and homogenized in 90 mL of buffered peptone water (BPW; HiMedia, India) diluted to 0.1% strength for initial enrichment and incubated aerobically overnight at 37°C. The enriched culture was streaked onto mannitol salt agar (Becton Dickinson, USA) and incubated at 37°C for 24 h. Presumptive *M. sciuri* colonies were confirmed by Gram staining (gram-positive cocci), catalase-positive, coagulase-negative, and oxidase-negative reactions, and the species level identity was further validated by whole-genome-based taxonomy using ANI analysis and TYGS-based digital DNA-DNA hybridization (8). Antimicrobial susceptibility was assessed by disk diffusion method and interpreted according to the latest CLSI 2025 guidelines. The isolate was identified as multidrug-resistant (MDR) (9). The isolate was sub-cultured until a pure single colony was obtained from the primary culture, then it was inoculated into 3 mL nutrient broth and incubated overnight at 37°C. Genomic DNA was extracted using the QIAamp DNA Mini Kit (cat. no. 51304, Qiagen GmbH), which involves cell lysis, DNA binding to a silica membrane, washing, and elution according to the manufacturer’s protocol. Sequencing libraries were prepared with Illumina TruSeq adapters, and paired-end sequencing (PE150, 50×) was performed on the Illumina NextSeq 2000, generating ~2.35 million reads. Raw reads were quality-checked with FastQC v0.11.5 (10) and trimmed using Fastp v0.23.2 (11). High-quality reads were assembled *de novo* using Unicycler v. v0.4.9 (12), and assembly quality was evaluated with QUAST v5.3.0 (13). Genome annotation was conducted with Prokka v1.14.6 (14) and refined using NCBI PGAP v6.10 (15). Genome quality assessment was done using CheckM v1.2.3 (16). Antibiotic resistance genes (ARGs) were identified by CARD (17) and ResFinder (18) and for virulence gene detection, VFDB (Virulence Factor Database, accessed on [October 19, 2025]) (19) was used via ABricate (https://github.com/tseemann/abricate). The pathogenicity index, plasmids, bacteriocin, and metabolic subsystems were predicted using PathogenFinder v2.0.0 (20), PlasmidFinder v2.1 (21), BAGEL4 (22), and RAST v2.0 (23) respectively. Unless otherwise noted, default parameters were used for all software.

The genomic characteristics of *Mammaliicoccus sciuri* BAU-MFRK-Mam-2025 are summarized in Table 1. The draft genome comprised 20 contigs, with a total size of 2,745,213 bp, GC content of 32.4%, and genome coverage of 50×. A pathogenicity score of 0.684 with five matched pathogenic families, highlighting its possible zoonotic importance that could be transmitted from food source.

**TABLE 1:** Genomic features of *Mammaliicoccus sciuri* strain BAU-MFRK-Mam-2025

Attributes	Values
GenBank accession	JBRKBH000000000
BioSample accession	SAMN51766299
BioProject	PRJNA1333650
SRA accession	SRR35580477
Genome size (bp)	2,745,213
GC content (%)	32.4%
Genome completeness (%)	99.5%
Contamination	2.4%
Number of contigs ≥500 bp	16
Number of contigs (#, ≥1,000 bp)	15
Largest contig (bp)	653,607
N50 value (bp)	447,243
Sequencing depth (×)	~227×
Average read length (bp)	150
Total bases generated (bp)	705,815,700
Genes (total)	2,772
CDSs (total)	2,714
Genes (coding)	2,697
CDSs (with protein)	2,697
Average nucleotide identity (ANI) (%)	98.83%
Compare to Refseq:	GCF_900474615.1_40295_D02_genomic.fna
rRNAs	1, 2, 1 (5S, 16S, 23S)
tRNAs	50
Genes (RNA)	58
tmRNA	1
Pseudo genes (total)	17
CDSs (without protein)	17
Complete rRNAs	1, 1 (5S, 23S)
Partial rRNAs	2 (16S)
Number of ARGs	6
Number of bacteriocins	2
Number of subsystems	259
Pathogenicity Score	0.684
Matched Pathogenic Families	5

## Data Availability

The draft genome sequence of *Mammaliicoccus sciuri* strain BAU-MFRK-Mam-2025 has been deposited in GenBank under accession number JBRKBH000000000, with BioSample accession SAMN51766299 and BioProject accession PRJNA1333650. Raw sequencing reads are available in the NCBI SRA database under accession number SRR35580477.
